# Retained placenta and postpartum hemorrhage: time is not everything

**DOI:** 10.1007/s00404-021-06027-5

**Published:** 2021-03-20

**Authors:** Denise Franke, Julia Zepf, Tilo Burkhardt, Philipp Stein, Roland Zimmermann, Christian Haslinger

**Affiliations:** 1 Department of Obstetrics, University Hospital Zurich, Frauenklinikstrasse 10, 8091 Zurich, Switzerland; 2 University of Zurich, Zurich, Switzerland; 3 Institute of Anesthesiology, Cantonal Hospital Winterthur, Winterthur, Switzerland

**Keywords:** Retained placenta, Postpartum hemorrhage, Third stage of labor, Uterine atony, Manual removal of the placenta

## Abstract

**Purpose:**

Postpartum hemorrhage is the major cause of maternal mortality worldwide. Retained placenta accounts for nearly 20% of severe cases. We investigated the influence of the time factor and retained placenta etiology on postpartum hemorrhage dynamics.

**Methods:**

Our retrospective study analyzed a single-center cohort of 296 women with retained placenta. Blood loss was measured using a validated and accurate technique based on calibrated blood collection bags, backed by the post- vs pre-partum decrease in hemoglobin. We evaluated the relationship between these two blood loss parameters and the duration of the third stage of labor using Spearman rank correlation, followed by subgroup analysis stratified by third stage duration and retained placenta etiology.

**Results:**

Correlation analysis revealed no association between third stage duration and measured blood loss or decrease in hemoglobin. A shorter third stage (< 60 min) was associated with significantly increased uterine atony (*p* = 0.001) and need for blood transfusion (*p* = 0.006). Uterine atony was significantly associated with greater decrease in hemoglobin (*p* < 0.001), higher measured blood loss (*p* < 0.001), postpartum hemorrhage (*p* = 0.048), and need for blood transfusion (*p* < 0.001).

**Conclusion:**

Postpartum blood loss does not correlate with third stage duration in women with retained placenta. Our results suggest that there is neither a safe time window preceding postpartum hemorrhage, nor justification for an early cut-off for manual removal of the placenta. The prompt detection of uterine atony and immediate prerequisites for manual removal of the placenta are key factors in the management of postpartum hemorrhage.

## Introduction

Postpartum hemorrhage (PPH) is the leading cause of maternal mortality worldwide: in 2003–2009, hemorrhage accounted for 27.1% of maternal deaths, over two-thirds of which were classified as PPH [1]. Etiologies include uterine atony, cervical or vaginal laceration, coagulopathy, and in 19.8% of cases retained placenta [2–5].

The third stage of labor starts after the delivery of the child and ends with the delivery of the placenta. A placenta is deemed retained if not expelled within 30 min postpartum [6, 7]. Prevalence is higher in high- vs low-income countries (2.7% vs 1.5%) [8]. Expulsion is rapid after vaginal delivery [9]. 90% of placentas deliver spontaneously within 15 min [10] (9 min in active management [11]); only 2.2% remain undelivered at 30 min [10].

Divergence persists over the optimal timing for manual removal of the placenta (MROP): ≥ 60 min in Northern Europe vs ≤ 30 min in Central and Southern Europe [12]. Evidence favoring MROP within 30 min, based on increasing PPH risk with the duration of the third stage of labor, emerged from studies combining women with normal and prolonged third stages [6, 9–11, 13–16]. However, the vast preponderance of normal over prolonged third stages may have skewed the correlation analysis of blood loss over time, thus weakening the argument for early MROP, an invasive and potentially unnecessary procedure with attendant risks.

We therefore opted, unlike previous studies, to evaluate the relationship between postpartum blood loss and third stage duration in a cohort confined exclusively to women with retained placenta. We aimed to investigate the influence of time and etiology of retained placenta on PPH, characterize the clinical course of women with retained placenta, and investigate the etiology underlying the pattern of blood loss.

## Materials and methods

The retrospective single-center cohort study was conducted at the University Hospital of Zurich following approval by the local institutional review board (ref. KEK-ZH 2016-1437, October 27 2016). Between January 2009 and December 2016 a total of 9058 births have taken place at the University Hospital of Zurich. 296 women with retained placenta after vaginal delivery of a ≥ 30-week fetus have been identified and included in our study. This cut-off for gestational age as inclusion criteria was chosen due to the high prevalence of retained placenta in deliveries at an earlier gestational age with a consecutive high risk for confounding factors regarding the primary objective of the study [10, 17]. The placenta was deemed retained if not expelled within 30 min postpartum. Non-inclusion criteria were preexisting coagulopathy and hepatic impairment affecting the prothrombin time.

### Data collection

Using our electronic databases (IntelliSpace Perinatal [OB TraceVue, https://www.usa.philips.com], Perinat, and KISIM [both University Hospital Zurich products]), two experienced clinical researchers (DF and JZ) manually extracted the relevant data on pregnancy course, delivery, maternal and neonatal outcome, perinatal diagnoses and interventions, together with the following primary outcome parameters: pre- and postpartum hemoglobin (g/l), measured blood loss (MBL, ml), PPH, and need for blood transfusion. Data were also collected on risk factors for increased blood loss (uterine atony, uterine leiomyoma, obstetric laceration, duration of the second and third stages of labor, operative vaginal delivery, previous cesarean delivery, suspected morbidly adherent placenta), feto-maternal characteristics (fetal weight [g], fetal head circumference [cm], gestational age [days], maternal body mass index [kg/m^2^, calculated either pre-pregnancy or in the first trimester], maternal age [years], multiple fetus pregnancy, parity, induction of labor, hypertension), and postpartum maternal anesthesia (general/regional, admission to intensive care). Gestational age was assessed by first-trimester ultrasonography or, rarely, by calculation from the first day of the last menstrual period.

### Management of the third stage of labor

The third stage of labor was actively managed in all cases after vaginal delivery. Oxytocin 5 IU was routinely given in a 5-min intravenous infusion immediately postpartum [18]. Controlled cord traction was performed. Physical examination by palpating the consistency of uterus was carried out regularly during the third stage of labor by the midwife and the attending physician. Also after the delivery of the placenta, a regular physical examination was performed (in the first hour postpartum every 15 min, in the second hour postpartum every 30 min, afterwards every 4 h). In case of suspected retained placenta, ultrasonography was performed and Credé's maneuver was performed only if ultrasonography showed the placenta to be trapped after detaching from the uterine wall. If the placenta did not detach after 15 min, oxytocin infusion was repeated. The anesthesiologist was called if the third stage persisted beyond 30 min or if MBL exceeded 300 ml at any time. The timing of the different interventions was recorded in the clinical information system and added to our database.

### Blood loss parameters

A fresh drape was placed under the mother immediately after vaginal delivery to quantify blood loss. Successive drapes were weighed on the calibrated neonatal scale present in each delivery suite. A graduated blood collection bag was placed under the mother before MROP or whenever MBL exceeded 300 ml and the placenta had still not detached.

Hemoglobin levels were obtained on admission and between 24 and 48 h postpartum to calculate the decrease in hemoglobin (∆-hemoglobin, g/l).

According to the WHO, PPH was defined as blood loss exceeding 500 ml in 24 h. [7, 19]

### Manual removal of the placenta

MROP was performed on the obstetric ward after anesthesia by manual exploration of the uterine cavity. Suspected remaining tissue was treated by ultrasound-guided curettage and all women received ceftriaxone 1 g intravenously. Blood loss was totaled at the end of the procedure. When atonic PPH was detected immediate MROP was indicated. In non-actively bleeding women with retained placenta, MROP was indicated after 30 min. From the point of indication (30 min postpartum in the non-bleeding patient) the anesthesiologist was called and everything was prepared for MROP.

### Statistical analysis

Baseline characteristics were reported using descriptive statistics.

We used Spearman’s rank correlation to analyze the association between duration of the third stage of labor and blood loss parameters (MBL and ∆-Hb).

The results prompted two subgroup analyses. First, we compared characteristics between women with a third stage shorter vs longer than 60 min. As explained above, our hospital policy required MROP and the presence of an anesthesiologist if the placenta was retained beyond 30 min, assuming no increase in bleeding. We considered the preparation time for anesthesia and further logistics and hence chose a 60 min cut off for third stage duration. Second, having observed a significant difference in the incidence of uterine atony (shown by our data to be the main risk factor for increased postpartum blood loss in women with retained placenta), we compared women with uterine atony and those without.

In the subgroup analyses, we used Fisher’s exact test for categorical variables and the Mann–Whitney *U* test for continuous variables. To investigate the relationship between MBL and each subgroup (third stage < 60 vs ≥ 60 min, and patients with vs without uterine atony), we used Spearman’s rank correlation analysis. A *p* value of < 0.05 was deemed significant throughout. All analyses were performed using STATA version 12.1 (STATA Corp., TX, USA).

## Results

**Fig. 1:**
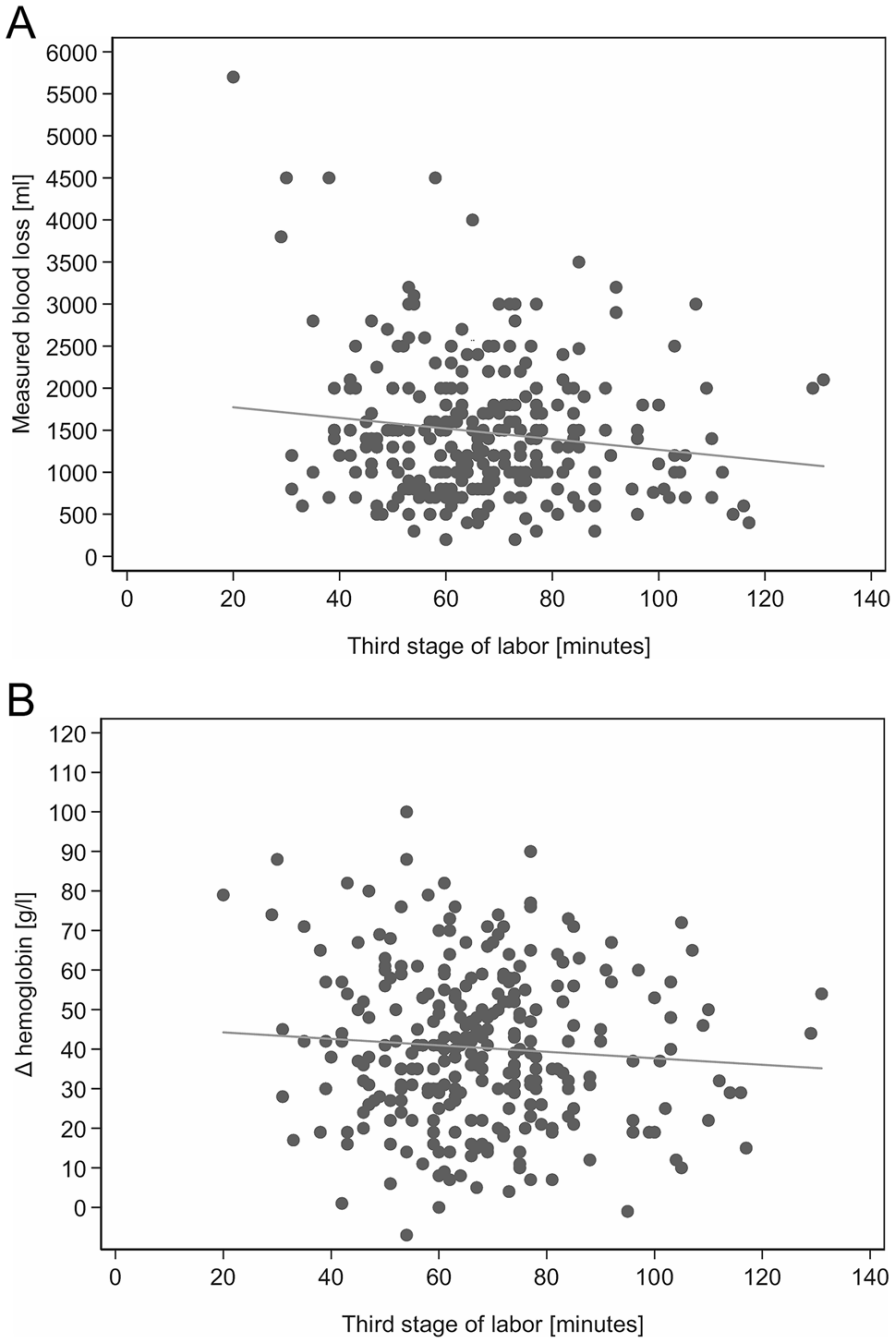
**a** Correlation of duration of the third stage of labor with MBL (ml), all patients (*n* = 296), *r* = −0.06, *p* = 0.311. **b** Correlation of duration of the third stage of labor with ∆-hemoglobin (g/l), all patients (*n* = 296), *r* = −0.04, *p* = 0.497

The characteristics, risk factors and outcome parameters in the 296 women (Table 1) showed PPH in 96.6% of patients, mean MBL 1300 ml (interquartile range [IQR] 900–1900 ml), and mean ∆-Hb 39 g/l (IQR 26–54 g/l); 4.5% of women needed blood transfusion. Correlation analysis, presented as scatterplots, showed no increase in MBL (*r* = −0.06, *p* = 0.311; Fig. 1a) or decrease in hemoglobin (*r* = −0.04, *p* = 0.497; Fig. 1b) with third stage duration.

**Table 1:** Characteristics and outcome parameters of all women with retained placenta

Variables	All *n* = 296
Outcome parameters	
Δ-Hemoglobin (g/l)^a^	39 (26–54%)
MBL (ml)	1300 (900–1900)
PPH (≥ 500 ml)	286 (96.6%)
Blood transfusion	13 (4.5%)
Risk factors for increased blood loss	
Uterine atony	81 (27.4%)
Uterine myomas	10 (3.4%)
BL due to maternal obstetric injuries^b^	16 (5.4%)
Second stage labor (min)	66 (21–124.5)
Second stage labor > 2 h	77 (26.0%)
Third stage of labor (min)	66 (55–77)
Operative vaginal delivery	46 (15.5%)
Previous cesarean section	16 (5.4%)
Morbidly adherent placenta	42 (14.2%)
Feto-maternal characteristics	
Fetal weight (g)	3230 (2805–3595)
Fetal head circumference (cm)	34.5 (33–35.5)
GA (d)	276.5 (262–284)
Maternal body mass index first trimester (kg/m^2^)	21.5 (19.85–24.6)
Maternal age (y)	33.8 (30.5–36.6)
Multiple fetus pregnancy	9 (3.0%)
Multiparity	129 (43.6%)
Primiparity	167 (56.4%)
Fetal weight ≥ 4000 g	16 (5.41%)
Induction of labor	85 (28.7%)
Hypertensive disorders^c^	12 (4.1%)
Anesthetic characteristics	
General anesthesia	52 (17.6%)
Regional anesthesia pp	136 (46.0%)
Admission to ICU pp	6 (2.0%)

Data are median (interquartile range) or *n* (%)

*MBL* measured blood loss; *PPH* postpartum hemorrhage; *GA* gestational age; *pp* postpartum; *EDA* epidural anesthesia; *ICU* intensive care unit

^a^Δ-Hemoglobin (g/l): difference in hemoglobin levels antepartum minus postpartum

^b^Maternal obstetric injuries: cervical, vaginal or perineal tears

^c^Hypertensive disorders include: prior existing hypertension, gestational-induced hypertension, preeclampsia and HELLP

Likewise, the first subgroup analysis (third stage < 60 min vs ≥ 60 min, Table 2) revealed no significant differences in MBL, ∆-Hb or frequency of PPH. In the shorter third stage subgroup, differences were significant in the need for blood transfusion (9.8% vs 1.9%, *p* = 0.006), presence of uterine atony (39.8% vs 21.2%, *p* = 0.001), frequency of operative vaginal delivery (24.7% vs 11.3%, *p* = 0.003), fetal head circumference (35 cm vs 34.5 cm, *p* = 0.049), frequency of multiple fetus pregnancy (6.5% vs 1.5%, *p* = 0.03), and postpartum regional anesthesia (18.3% vs 58.6%, *p* < 0.001).

**Table 2:** Subgroup analysis on the duration of the third stage of labor (< 60 min; > 60 min)

Variables	Third stage of labor < 60 min (*n* = 93)	Third stage of labor ≥ 60 min (*n* = 203)	*p* value
Outcome parameters			
Δ-Hemoglobin^a^ (g/l)	38 (28–56)	39 (25–54)	0.676
MBL (ml)	1400 (900–2000)	1300 (900–1800)	0.301
PPH (≥ 500 ml)	92 (98.9%)	194 (95.6%)	0.124
Blood transfusion	9 (9.8%)	4 (1.9%)	0.006
Risk factors for increased blood loss			
Uterine atony	37 (39.8%)	44 (21.2%)	0.001
Uterine myomas	5 (5.4%)	5 (2.5%)	0.172
BL due to maternal obstetric injuries^b^	3 (3.2%)	13 (6.4%)	0.199
Second stage labor (min)	87 (27–155)	47 (20–119)	0.058
Second stage labor > 2 h	30 (32.3%)	47(23.2%)	0.097
Third stage of labor (min)	51 (45–55)	73 (66–82)	< 0.001
Operative vaginal delivery	23 (24.7%)	23 (11.3%)	0.003
Previous cesarean section	7 (7.5%)	9 (4.4%)	0.275
Morbidly adherent placenta	15 (16.1%)	27 (13.3%)	0.517
Feto-maternal characteristics			
Fetal weight (g)	3300 (2900–3680)	3190 (2770–3560)	0.091
Fetal head circumference (cm)	35 (33.5–36)	34.5 (33–35.5)	0.049
GA (d)	276 (267–285)	277 (261–284)	0.770
Maternal body mass index first trimester (kg/m^2^)	21.4 (20.3–24.1)	21.5 (19.8–24.9)	0.859
Maternal age (y)	33.54 (29.9–36.44)	34.08 (30.52–36.66)	0.561
Multiple fetus pregnancy	6 (6.5%)	3 (1.5%)	0.030
Multiparity	41 (44.1%)	88 (43.4%)	0.502
Primiparity	52 (55.9%)	115 (56.7%)	0.498
Fetal weight ≥ 4000 g	8 (8.6%)	8 (3.9%)	0.088
Induction of labor	26 (28.0%)	59 (29.2%)	0.480
Hypertensive disorders^c^	3 (3.2%)	9 (4.4%)	0.447
Anesthetic characteristics			
General anesthesia	21 (22.6%)	31 (15.3%)	0.125
Regional anesthesia pp	17 (18.3%)	119 (58.6%)	< 0.001
Admission to ICU pp	4 (4.3%)	2 (1.0%)	0.060

Data are median (interquartile range) or *n* (%)

*MBL* measured blood loss; *PPH* postpartum hemorrhage; *GA* gestational age; *pp* postpartum; *EDA* epidural anesthesia; *ICU* intensive care unit

^a^Δ-Hemoglobin (g/l): difference in hemoglobin levels antepartum minus postpartum

^b^Maternal obstetric injuries: cervical, vaginal or perineal tears

^c^Hypertensive disorders include: prior existing hypertension, gestational-induced hypertension, preeclampsia and HELLP

The second subgroup analysis (Table 3) revealed that women with uterine atony were significantly more likely to experience a greater decrease in hemoglobin (55 g/l vs 35 g/l, *p* < 0.001), increased MBL (2000 ml vs 1100 ml, *p* < 0.001), frequency of PPH (100% vs 95.4%, *p* = 0.048), and need for blood transfusion (13.6% vs 0.9%, *p* < 0.001). This group was also more likely to have a shorter third stage (61.5 min vs 67 min, *p* = 0.01), require general anesthesia (39.5% vs 9.3%, *p* < 0.001), and be admitted to intensive care postpartum (6.2% vs 0.5%, *p* = 0.002).

**Table 3:** Patients with retained placenta with or without uterine atony

Variables	Uterine atony (*n* = 81)	No uterine atony (*n* = 215)	*p* value
Outcome parameters			
Δ-Hemoglobin (g/l) ^a^	55 (39–67.5)	35 (22–47)	< 0.001
MBL (ml)	2000 (1500–2600)	1100 (800–1600)	< 0.001
PPH (≥ 500 ml)	81 (100.0%)	205 (95.4%)	0.048
Blood transfusion	11 (13.6%)	2 (0.9%)	< 0.001
Risk factors for increased blood loss			
Uterine myomas	2 (2.5%)	8 (3.7%)	0.595
BL due to maternal obstetric ^b^ injuries	4 (4.9%)	12 (5.6%)	0.821
Second stage labor (min)	69 (21–127)	60 (21–122)	0.814
Second stage labor > 2 h	23 (28.4%)	54 (25.1%)	0.566
Third stage of labor (min)	61.5 (50–73.5)	67 (59–77)	0.010
Operative vaginal delivery	13 (16.1%)	33 (15.4%)	0.882
Previous caesarean section	7 (8.6%)	9 (4.2%)	0.131
Morbidly adherent placenta	14 (17.3%)	28 (13.0%)	0.349
Feto-maternal characteristics			
Fetal weight (g)	3240 (2900–3690)	3220 (2760–3570)	0.264
Fetal head circumference (cm)	34.5 (33.5–36)	34.5 (33–35.5)	0.307
GA (d)	278 (269–284)	276 (262–284)	0.381
Maternal body mass index first trimester (kg/m^2^)	21.3 (20.1–24.7)	21.5 (19.8–24.5)	0.957
Maternal age (y)	34.5 (31.7–37.2)	33.6 (30.2–36.5)	0.248
Multiple fetus pregnancy	3 (3.7%)	6 (2.8%)	0.683
Multiparity	38 (46.9%)	91 (42.3%)	0.478
Primiparity	43 (53.1%)	124 (57.7%)	0.522
Fetal weight ≥ 4000 g	4 (4.9%)	12 (5.6%)	0.827
Induction of labor	28 (34.6%)	57 (26.5%)	0.172
Hypertensive disorders^c^	6 (7.4%)	6 (2.8%)	0.073
Anesthetic characteristics			
General anesthesia	32 (39.5%)	20 (9.3%)	< 0.001
Regional anesthesia pp	26 (32.1%)	110 (51.2%)	0.003
Admission to ICU postpartum	5 (6.2%)	1 (0.5%)	0.002

Data are median (interquartile range) or *n* (%)

*MBL* measured blood loss; *PPH* postpartum hemorrhage; *GA* gestational age; *pp* postpartum; *EDA* epidural anesthesia; *ICU* intensive care unit

^a^Δ-Hb (g/l): difference in hemoglobin levels antepartum minus postpartum

^b^Maternal obstetric injuries: cervical, vaginal or perineal tears

^c^Hypertensive disorders include: prior existing hypertension, gestational induced hypertension, preeclampsia and HELLP

**Fig. 2:**
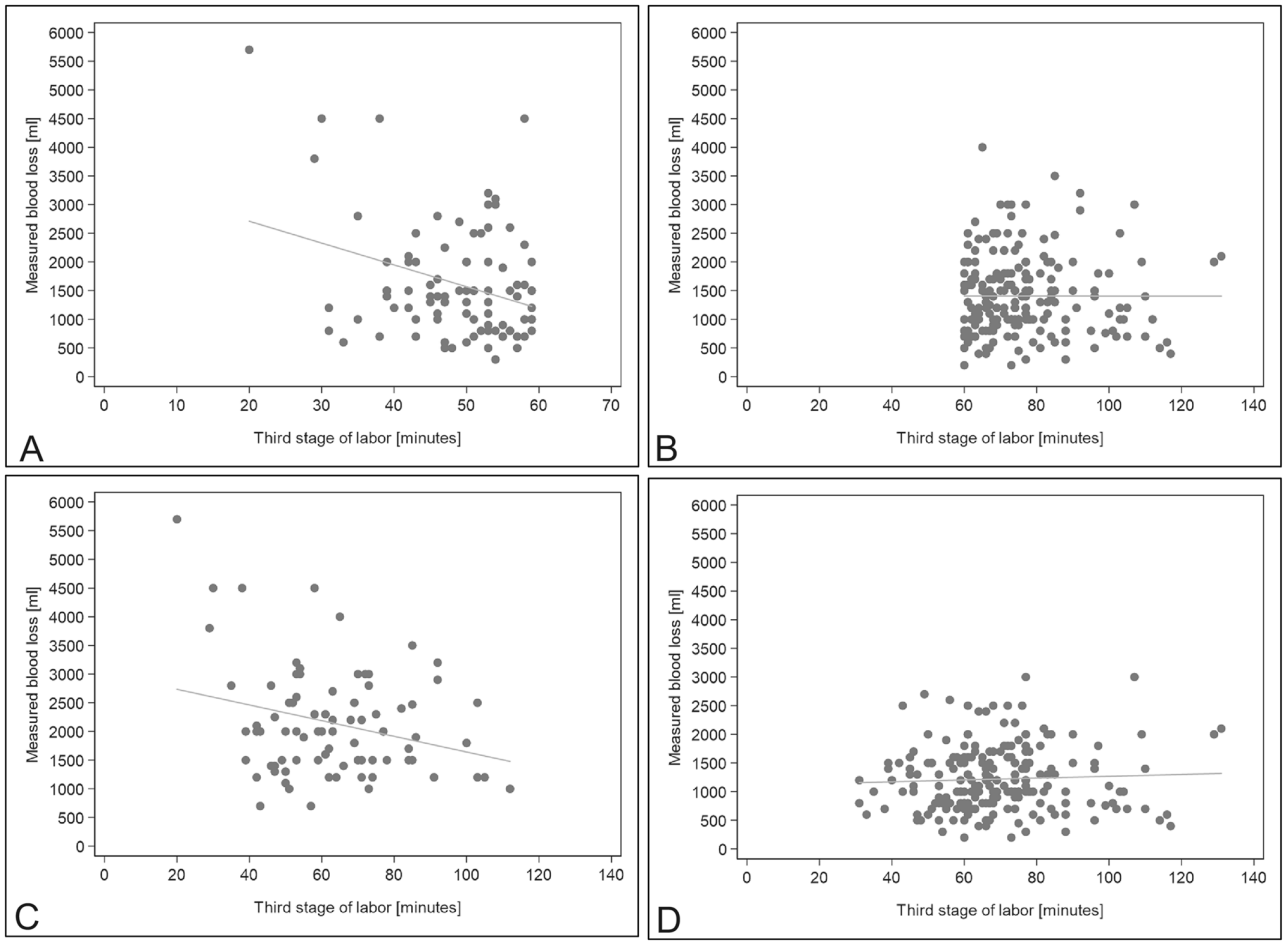
**a–d** Correlation analysis between the duration of the third stage of labor and measured blood loss (MBL) (ml) of: **a** patients with a third stage of labor < 60 min, **b** patients with a third stage of labor ≥ 60 min, **c** patients with uterine atony, **d** patients without uterine atony). **a** Correlation of duration of the third stage of labor with MBL (ml), in patients with third stage duration < 60 min (*n* = 93), *r* = −0.14, *p* = 0.178. **b** Correlation of duration of the third stage of labor with MBL (ml), in patients with third stage duration ≥ 60 min (*n* = 203), *r* = −0.14, *p* = 0.178. **c** Correlation of duration of the third stage of labor with MBL (ml), in patients with uterine atony (*n* = 81), *r* = −0.06, *p* = 0.311. **d** Correlation of duration of the third stage of labor with MBL (ml), in patients without uterine atony (*n* = 215), *r* = 0.05, *p* = 0.470

As in the overall population, Spearman analysis found no significant correlation between MBL and third stage duration in all four subgroups (Fig. 2a–d).

## Discussion

The association between postpartum blood loss and retained placenta, or duration of the third stage of labor, is a major obstetric concern. Consensus on an optimal MROP cut-off remains elusive [2–4, 6–9, 11, 12, 20]. Yet to our knowledge, the dynamics of blood loss over time had not previously been studied in a cohort confined to women with retained placenta. Our study with this population yielded no evidence for increasing blood loss with third stage duration. No definite MROP cut-off can thus be set.

Our finding conflicts with several earlier studies. Apparently, increased blood loss over time prompted proposals to end the third stage at 30 min [6, 10]. A 2008 review extended the cut-off to 30–60 min [20], while more recent studies even shortened it to 10–20 min [9, 11, 13, 15, 16].

However, these recommendations were derived from cohorts mainly comprising normal-duration third stages. Blanket cut-offs disregard retained placenta etiology [6, 9–11, 13–16], and clinical status. Given a mean 5.5 min for normal placenta delivery with active management [11], a preponderance of such cases could skew the analysis into suggesting that blood loss increases over time. In our population, no blood loss increase exists if the analysis is confined to women with retained placenta: blood loss in the third stage even decreases slightly and no difference over time is observed (Fig. 1). Our results are endorsed by another cohort study, which found that retained placenta as diagnosis itself was a strong predictor of quantity of blood loss, but that the bare duration of the third stage of labor, as in our study, was a weak predictor of quantity of blood loss [21]. Our findings suggest a pattern of blood loss dependent more on retained placenta etiology than on time. The frequency of uterine atony was 27.4%, consistent with the increasing frequency of atonic PPH [22]. Our data support an association between shorter third stage of labor and uterine atony, characterized by immediate severe PPH, hence significantly increased blood loss, decreased hemoglobin, and need for blood transfusion (Tables 2 and 3). Severity is underlined by significantly more admission to intensive care and need for general anesthesia. Thus, blood loss dynamics in uterine atony may partly explain the divergence between our results and those of previous researchers. Certainly, we cannot conclude that uterine atony would lead to a shorter third stage of labor or that shorter third stage of labor might be associated with uterine atony. Actually, we presume that uterine atony led to increased blood loss and consecutively to an earlier indication for MROP—irrespective of the time since delivery. A finding that underlines the importance of a close monitoring of women after the delivery of their child.

Only one randomized controlled trial has compared differing MROP cut-offs (10 vs 15 min): it reported decreased hemodynamic compromise using 10 min [15]. Nevertheless, MROP is invasive, with its own risks, including increased blood loss and need for transfusion [10, 16, 23]. An early bonding and a positive birth experience may appear secondary considerations compared to the severity of PPH, but they must be factored into decision making given the multiple MROP interventions needed to prevent one case of hemodynamic compromise [24–26].

Hence, our reluctance to advocate blanket early MROP for retained placenta regardless of underlying etiology or clinical status before 30 min postpartum. Instead, we propose continuous third-stage monitoring for early signs of uterine atony or increased blood loss. Visual observation and vital parameter surveillance should be complemented by regular fundal palpation to assess tone and by ultrasonography to detect morbidly adherent placenta [27], detached but trapped placenta, and massive blood pooling in the atonic uterus without visible bleeding. Obstetric and anesthetist teams must be ready for MROP at any time should bleeding increase, irrespective of preset cut-offs. No safe time window exists, however early it is set.

We based our study on data manually collected by two experienced investigators (DF and JZ), double-checked by a third investigator (CH). We used anesthetic protocols and obstetrician input to verify third-stage duration data. As for blood loss measurement—justifiably reported as error-prone [28–30]—the real-time technique used in every woman was developed in-house specifically to manage PPH; feasibility and accuracy in the clinical setting were recently validated in 921 women [31].

Possible methodological limitations include retrospective data evaluation, making precise etiology impossible to determine. For example, we do not have data regarding labor augmentation with oxytocin during the second stage of labor and its effect on PPH. Furthermore, the single-center design may cause selection bias, although ensuring that all women were treated by the same protocol. We cannot know whether atony caused a fully or partially retained placenta with immediately increased blood loss or whether atony with blood loss was observed only after MROP. However, there was no correlation between MBL and third stage duration in either the overall population or any subgroup. A further limitation is the possibly underestimated frequency of morbidly adherent placenta: placental histology was only requested if the obstetrician performing MROP clinically suspected the diagnosis. Furthermore, this diagnosis is most reliable only in case of hysterectomy with histopathological follow-up (*n* = 3).

## Conclusion

In summary, we found no evidence for increased blood loss over time in the third stage. We therefore cannot identify a safe time window. Our analysis indicates that women with atonic retained placenta risk immediate severe hemorrhage and have a significant higher need for blood transfusion. Delay can only compound blood loss. Prompt diagnosis followed by emergency MROP is required regardless of set cut-offs. In retained placenta with no increased blood loss in the non-atonic patient, a blanket policy of early cut-off could constitute overtreatment, hence our reluctance to recommend MROP before 30 min postpartum. We rather promote a close monitoring of the woman and a well-organized setting to be able to react promptly.
